# A209 ASSESSMENT OF OUTCOMES OF PATIENTS ADMITTED WITH ACUTE, SEVERE ULCERATIVE COLITIS ON ESTABLISHED BIOLOGIC THERAPY: A SINGLE CENTRE RETROSPECTIVE ANALYSIS

**DOI:** 10.1093/jcag/gwac036.209

**Published:** 2023-03-07

**Authors:** N Sabrie, M Jogendran, R Jogendran, L Targownik

**Affiliations:** 1 University of Toronto, Toronto; 2 Queen's University, Kingston; 3 Gastroenterology, University of Toronto; 4 Gastroenterology, Mount Sinai Hospital, Toronto, Canada

## Abstract

**Background:**

Acute, severe, ulcerative colitis (ASUC) is associated with a high morbidity and mortality. Current guidelines recommend the initiation of high dose intravenous steroids, followed by anti-TNF therapy if a satisfactory therapeutic response is not rapidly achieved. However, guidelines are agnostic on how to manage patients who are admitted for ASUC despite being on established biologic therapy. Furthermore, short-term clinical outcomes in this population are not well characterized.

**Purpose:**

The aim of this study is to assess the differences in short-term clinical outcomes in patients admitted with ASUC who are on established biologic therapy compared to those not on established biologic therapy.

**Method:**

We conducted a retrospective chart review of patients admitted with ASUC to Mount Sinai Hospital (MSH) in Toronto, Ontario from January 2018 until December 2021. Patients were included if they were deemed to have a severe flare, defined as having 6 or more loose bowel movements per day with at least one of the following features: temperature of 38.0 Celsius, tachycardia, anemia, or elevated inflammatory markers. Included subjects were considered to be on established biologic therapy if they had a biologic within 56 days prior to admission, all other admitted subjects were included as controls. Our primary outcome was the difference in hospital length of stay (HLOS). We also contrasted duration of intravenous steroids, rates of surgical consultation, rates of in-hospital colectomy, and readmission rates within 90 days of discharge.

**Result(s):**

130 charts were included in our study, 53 of which were patients on established biologic therapy, and 77 of which were patients not on established biologic therapy. The HLOS between the two groups was not significantly different, (7.23 days [established biologic therapy] vs.7.47 days [not on biologic therapy], p value = 0.77). Patients on established biologic therapy were more likely to receive an inpatient surgical consultation (33.96% vs 7.79%, p-value <0.001). However, rates of colectomy prior to discharge were not statistically different (1.89% vs 0%, p-value = 0.23). Patients on established biologic therapy were significantly more likely to be readmitted within 90 days of discharge (30.19% vs 12.99%, p-value = 0.016).

**Image:**

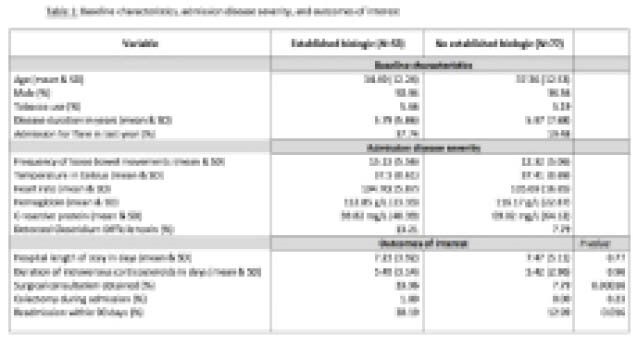

**Conclusion(s):**

Although there were no differences in HLOS and colectomy rates between the 2 groups, patients with ASUC on established biologic therapy were more likely to be readmitted within 3 months of discharge. Further work is required to define optimal medical management of persons admitted with ASUC who are failing biologic therapies.

**Please acknowledge all funding agencies by checking the applicable boxes below:**

None

**Disclosure of Interest:**

None Declared

